# An Improved Passivity-based Control of Electrostatic MEMS Device

**DOI:** 10.3390/mi11070688

**Published:** 2020-07-16

**Authors:** Mutaz Ryalat, Hazem Salim Damiri, Hisham ElMoaqet, Imad AlRabadi

**Affiliations:** 1 Mechatronics Engineering Department, School of Applied Technical Sciences, German Jordanian University, Amman 11180, Jordan; Hisham.ElMoaqet@gju.edu.jo (H.E.); I.AlRabadi@gju.edu.jo (I.A.); 2 IEEE Member, Piscataway, NJ 08854, USA; Hazemdomeri@gmail.com

**Keywords:** Hamiltonian systems, MEMS, passivity-based control, pull-in stability, observer design

## Abstract

It is commonly known that the performance of an electrostatic microelectromechanical system (MEMS) device is limited to a specific range of the full gap distance due to the inherited “pull-in instability” phenomenon. In this work, we design a controller to extend the stabilization range of an electrostatic MEMS device and to enhance its performance. The interconnection and damping assignment-passivity based control (IDA-PBC) method is used and the controller design involves coordinate transformations and a coupling between the mechanical and electrical subsystems of the device. The method deploys a design of a speed observer to estimate the speed, which cannot be directly measured by sensors. The effectiveness of the dynamical controller is verified via numerical simulations; it is evident by the extended travel range of the parallel plates as well as the improved performance of the plates, even with a naturally lighter damping ratio.

## 1. Introduction

The electrostatic actuator is commonly used in microelectromechanical system (MEMS) applications such as microswitches [1], micromirrors [2], microvalves [3] and micropumps [4]. The actuator is characterized by the simplicity of its structure and the flexibility of the operation. However, the performance of the electrostatic MEMS actuator is limited to a specific range. If an open-loop control approach is used to control the position of the actuator, the stability is limited to only one third of the full gap distance, which is called the pull-in or snap-through range [5]. Therefore, the system suffers from instability issues by undergoing a saddle node bifurcation. Different advanced closed-loop control strategies were proposed in the literature to extend the travel range of the actuator.

A linearization method was deployed in some works, as in [6], while in others nonlinear control techniques were proposed, such as static and dynamic sliding mode control [7], backstepping control [8,9], and differential flatness [10]. Nonlinear control approaches based on the passivity-based control of MEMS have also been reported in the literature. In [11], this method has been used to stabilize a one-degree of freedom actuator in the presence of parasitic capacitance. Moreover, the passivity-based design has been proposed in [5] to stabilize and improve the performance of an electrostatic actuator. Other works focused on other issues associated with this type of MEMS including; eliminating the influence of the parasitic capacitance, which is a critical problem in MEMS capacitive accelerometers [12]. The influence of the applied driving force was studied to evaluate the measurement variations in resonant characteristics of electrostatically actuated MEMS resonators [13] and the influence of the surface processing technology on the mechanical properties and behaviors of the electrostatic MEMS devices [14].

In addition to the works which have focused on the stability and controller design methods for MEMS actuators, there have been several works reported in the literature investigating the design, fabrication, and characterization of the electrostatic MEMS devices, aiming at improving the modelling accuracy, counteracting the non-linear effects of pull-in instability to extend the range of operation, and to create devices that yield good performance even under changing operational conditions and environments [15]. Such works have dealt with the design, structure shape and geometry of the device (mainly the electrodes), as well as taking into consideration some practical considerations, such as the effect of fringing fields, deformation, buckling, nonlinear structural stiffness (spring configuration), and stiction/adhesion. A comprehensive review that includes several proposed approaches was reported in [1]. A noticeable structural design that solves the shortcomings of the parallel plates type (including pull-in instability, displacement limitation and high voltage demands) due to electrostatic spring softening nonlinearity is the curved electrodes (microbeams), arches or buckled beams [16]. The curved electrodes offer large displacements for lower actuation voltages and power dissipation. Additionally, the pull-in instability range is minimized, thus extending the stable region while maintaining large electrostatic forces [15,16,17,18]. The curved electrodes were successfully used in many applications reported in the literature, including fabricating curved silicon membranes. They demonstrated their utility as MEMS actuators [15], curved electrode electrostatic actuators in viscous dielectric media [16], for underwater bio-MEMS applications [17], and for microvalve applications [18].

One of the passivity-based methods that has recently been used in many important control applications is the interconnection and damping assignment passivity-based control (IDA-PBC) approach. This method was introduced in [19] to control port-Hamiltonian systems by damping injection and energy shaping. The IDA-PBC has been applied to different types of mechanical, electrical and mechatronic systems such as quadrotors [20], rotary inverted pendulums [21], full bridge rectifiers [22] and bond graphs [23]. In addition, this method was used in electrostatic MEMS actuators to improve the stability and the behavior of the system. In [24,25], the IDA-PBC was used to control and improve the performance of an electrostatic MEMS actuator which belongs to a class of weakly-coupled electromechanical systems. As thoroughly discussed in [25], those systems are characterized by a lack of effective coupling between the mechanical and electrical subsystems. By utilizing the coordinate transformations and introducing integral control along with damping injection, stabilization and performance enhancement as well as robustness of the system against disturbances are obtained. Additionally, designing the controller by coordinate transformations was also used in the studies [26,27]. In [25], a coupling was introduced in the interconnection matrix in a magnetic levitation system to enhance the coupling between the mechanical subsystem (the momentum coordinate) and the electrical subsystem (the flux coordinate). 

However, for the MEMS application, as it is difficult practically to involve speed measurements, the coupling that leads to the appearance of momentum (speed) states in the controller has been avoided. Motivated by these previous works and the wide applications of electrostatic-type MEMS, the main contribution of this paper is to analyze this system focusing on its inherently nonlinear effect called “pull-in instability”. We then introduce the dynamic IDA-PBC controller by enforcing a coupling between the momentum coordinate and the charge coordinate in an electrostatic MEMS actuator to improve the transient response of the system and extend its range of operation, i.e., beyond its pull-in instability range. This requires the construction of a speed observer to estimate the speed (momentum) of the PCH model, which is not achievable practically. 

The rest of the paper is organized as follows: In Section 2, the mathematical model of the electrostatic MEMS actuator is introduced. A full pull-in stability analysis of the system is discussed in Section 3. Section 4 presents the port-Hamiltonian model of the system and the design of a dynamic IDA-PBC controller along with the speed observer. The results of numerical simulations are shown in Section 5. Finally, Section 6 gives concluding remarks.

## 2. The Mathematical Model of the Electrostatic MEMS Actuator

**Figure 1:**
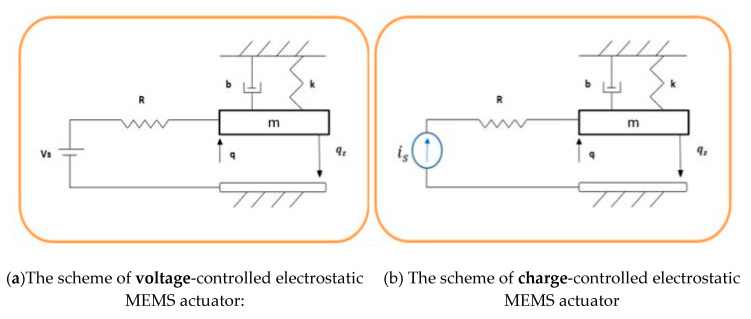
Scheme of 1-DOF parallel-plate electrostatic actuator.

The schematic of an electrostatic actuator is shown in Figure 1 The mechanical subsystem consists of a linear spring with stiffness k, a damper with damping coefficient b and a movable plate with mass m, while the other plate is fixed. The gap distance is q(t) and $\;q_{z}$ is the distance when the applied voltage is zero. The electrical subsystem consists of a resistor R and a voltage source $V_{s}$ to drive the system. The mechanical subsystem equation can be found by applying Newton’s second law to the mechanical parts [5,24]:

(1) $$m\ddot{q}\left(t\right)+b\dot{q}\left(t\right)+k\left(q\left(t\right)-q_{z}\right)=-F_{electric}$$

where $F_{electric}$ is the electrostatic force which can be given by:

(2a) $$F_{electric}=\frac{Q{\left(t\right)}^{2}}{2A\epsilon }$$

where Q(t) is the electric charge, A is the area of the moving plate and ϵ is the electrical permittivity. Using the actuator capacitance C=Aϵ/q and the relationship between the charge and voltage of the parallel plate capacitor Q=CV, the electrostatic force can be also represented using voltage representation as

(2b) $$F_{electric}=-\frac{A\epsilon V_{s}^{2}}{2q^{2}}$$

The electrical subsystem equation can be found by applying Kirchhoff’s voltage law to the circuit [5,24]:

(3) $$\dot{Q}\left(t\right)=\frac{1}{R}\left(V_{s}-\frac{Q\left(t\right)q\left(t\right)}{A\epsilon }\right)$$

The equations of the system can be found by rearranging (1–3) and defining the states vector as [q,p,Q]^T^ =$\left[x_{1},x_{2},x_{3}\right]$^T^ where *p* is the momentum. Thus, the equations can be rewritten as [24]:

(4a) $${\dot{x}}_{1}=\frac{x_{2}}{m}$$

(4b) $${\dot{x}}_{2}=-k\left(x_{1}-q_{z}\right)-\frac{bx_{2}}{m}-\frac{x_{3}^{2}}{2A\epsilon }$$

(4c) $${\dot{x}}_{3}=-\frac{x_{1}x_{3}}{\epsilon RA}+\frac{V_{s}}{R}$$

In Equation (4c), $V_{s}$ is the control input u. The position of the movable plate is controlled typically via a voltage or charge control depending on the type of the electrical parameter (source) being applied across the parallel plates (capacitor). A voltage-controlled electrostatic MEMS (Figure 1a) uses a voltage source, while for a charged-controlled electrostatic MEMS (Figure 1b) the electrical source is a current source that directly controls the amount of charge on the plates [28].

Here, we adopt the voltage-controlled method. Before proceeding with the analysis and controller design, the equilibrium points of the system must be defined. The equilibria can be estimated by setting (4) to zero, that is

(*a) $${\dot{x}}_{1}=\frac{x_{2}}{m}=0\;\Rightarrow \;x_{2}=0$$

(*b) $${\dot{x}}_{2}=-k\left(x_{1}-q_{z}\right)-\frac{bx_{2}}{m}-\frac{x_{3}^{2}}{2A\epsilon }=0\;\Rightarrow \;x_{3}^{2}=2Ak\epsilon (q_{z}-x_{1})$$

(*c) $${\dot{x}}_{3}=\frac{-x_{1}x_{3}}{\epsilon RA}+\frac{u}{R}=0\;\Rightarrow \;u=\frac{x_{1}x_{3}}{A\epsilon \;}$$

As discussed in Remark 3.1 and [29], in such a system, which is in fact an underdetermined system, the equilibria are called forced equilibria as the position of the plate can only be maintained by means of a bias voltage or drained current from the input source [30]. Therefore, for any given desired equilibrium (position) $x_{1d}\;\in \;\left(0,\;q_{z}\right)$ there is a corresponding desired input $u_{d}$.

For the charge-controlled method, it can also be shown from Equation (*), the equilibrium for a desired position $x_{1d}\;$, given $x_{2}=0$, $x_{3d}=\sqrt{2Ak\epsilon (q_{z}-x_{1d})\;}$ and $u_{d}=V_{s}{}_{d}=\frac{x_{1d}x_{3d}}{A\epsilon \;}$. When the system is at rest, the voltage $u_{d}=V_{sd}=0$, which implies the charge ($x_{3d}=0$) and from (*b)  $\Rightarrow \;x_{1d}=q_{z}$. For the case of full-gap position when $x_{1}=0$ and from (*b) $\Rightarrow \;x_{3d}=\sqrt{2Ak\epsilon q_{z}}$. The equilibrium for the voltage-controlled method is determined based on the pull-in stability phenomenon which will be discussed in Section 3.

**Remark** **2.1.** *Another main difference between the charge and voltage-controlled methods is the type of energy function used to define the generalized effort (electrostatic force) $F_{\mathrm{electric}}$ the energy function is used in the case of the charge-controlled method while the co-energy function is used in the voltage-controlled method [31].*

To avoid numerical problems, the equations can be normalized as in [5,7,21,24] by defining the states of the system as $\left[X_{1},X_{2},X_{3}\right]$ = [$\frac{x1}{\alpha }$, $\frac{\dot{x}1}{\alpha }$, $\frac{x3}{\beta }$], and α,β can be defined as [21]:

(5) α=ϵσAR

(6) $$\beta =\epsilon \sigma A\sqrt{\sigma mR}$$

(7) v=σRβ

where σ is the attenuation in the equation $t=\sigma \hat{t}$. The natural frequency $\omega_{n}$ and the damping ratio ζ can be defined as:

(8) $$\omega_{n}=\sqrt{\frac{k}{m}}$$

(9) $$2\zeta \omega_{n}=\frac{b}{m}$$

(10) $$\omega =\frac{\omega_{n}}{\sigma }$$

Using (5–10) the equations of the normalized system can be rewritten as [21]:

(11a) $${\dot{X}}_{1}=X_{2}$$

(11b) $${\dot{X}}_{2}=-2\zeta \omega X_{2}-\omega^{2}\left(X_{1}-q_{z}\right)-\frac{X_{3}^{2}}{2}$$

(11c) $${\dot{X}}_{3}=-X_{1}X_{3}+u.$$

## 3. The Pull-in Stability Analysis of the System

In this section we investigate the pull-in stability phenomena which is the main challenge in the design and control of the electrostatic MEMS as thoroughly discussed in [1,21,31].

The kinetic energy (KE) of the system is:

(12) $$K\left(x_{2}\right)=\frac{1}{2m}x_{2}{}^{2}$$

and the potential energy (PE) of the system comprises the elastic potential of the linear spring and the electrostatic energy of the capacitor subject to a voltage $V_{s}$:

(13) $$V\left(x_{1}\right)=\frac{1}{2}k\;{\left(x_{1}-q_{z}\right)}^{2}+\frac{A\epsilon V_{s}^{2}}{2x_{1}}=\frac{k}{2}{\left(x_{1}-q_{z}\right)}^{2}+\frac{x_{1}x_{3}^{2}}{2A\epsilon }$$

First, we introduce the following remark [25]:

**Remark** **3.1.** *At the desired equilibrium of the EM system, the electrical energy—electrostatic (stored in capacitor) or electromagnetic (stored in inductors)—is never zero as some current has to be drained from the source to keep the mechanical coordinate at some nonzero value [29]. Therefore, the input voltage $V_{s}$ determines the force, which stretches the spring, thus determines the change in gap. As $V_{s}$ increases, the charge $x_{3}$ also increases and the force of attraction between the plates increases, whereas the gap $x_{1}$ decreases, and thus requires the spring to be stretched from its rest position. Therefore, as shown in Figure 1, at the rest position we have u = $V_{s}$ = $x_{3}$ = 0, while the spring is unstretched, in which case $x_{1}$ = $q_{z}$. Moreover, the stabilization of this system at any point in the gap is achieved through the balance between the elastic restoring force k($x_{1}$ − $q_{z}$) and the electrostatic force $\;\frac{x_{3}^{2}}{2A\epsilon }$. Thus, at any forced equilibrium position $x_{1}$ = $x_{1qq}$ ∈ (0, $q_{z}$) [31], with $u(t)=V_{seq}\left(t\right)\;$ the following must hold:*

$$F_{spring}=F_{electric}\;$$

(14) $$k\left(x_{1eq}-q_{z}\right)=\frac{x_{3eq}^{2}}{2A\epsilon }=\frac{A\epsilon V_{seq}^{2}}{2x_{1eq}{}^{2}}$$

At some critical voltage when a displacement of the top plate is equal to one-third of the zero-voltage gap, the system becomes unstable and this movable top plate rapidly snaps down to the fixed bottom plate, i.e., the gap collapses to zero. This phenomenon is called “snapthrough” or “pull-in”. Pull-in instability is an inherently nonlinear and crucial undesirable effect. In this section, we perform stability analysis of the MEMS device, and we will show using energy (Hamiltonian) and linearization approaches why the equilibrium of this system is only stable within one-third of the nominal gap. The stability of this actuator is achieved through the balance between the elastic restoring force $k\left(x_{1}-q_{z}\right)$ and the electrostatic force (2), which must be equal at equilibrium position $x_{1eq}\;\in \;\left(0,\;q_{z}\right)$ as in (14). As discussed in [19], a Hamiltonian system is stable if the PE V (q) has an isolated minimum at the desired equilibrium:

(15) $$x_{1eq}=\arg\min V\left(q\right)\;$$

This is satisfied if both ${\nabla_{x_{1}}V|}_{x_{1}=x_{1eq}}=0$ and ${\nabla_{x_{1}}^{2}V|}_{x_{1}=x_{1eq}}>0$ hold. The total PE of the system comprises the elastic potential of the linear spring and the electrostatic energy

(16) $$V\left(x_{1}\right)=\frac{1}{2}k\;{\left(x_{1}-q_{z}\right)}^{2}+\frac{A\epsilon V_{seq}^{2}}{2x_{1}}$$

Substituting $V_{seq}^{2}$ from (14) into (13), we obtain:

(17) $$V\left(x_{1}\right)=\frac{1}{2}k\;{\left(x_{1}-q_{z}\right)}^{2}+\frac{kx_{1eq}^{2}\left(x_{1eq}-q_{z}\right)}{x_{1}}$$

Therefore,

(18) $${\nabla_{x_{1}}^{2}V|}_{x_{1}=x_{1eq}}=k\left(1+\frac{2\left(x_{1eq}-q_{z}\right)}{x_{1eq}}\right)=k\left(3-\frac{2q_{z}}{x_{1eq}}\right)>0\;\to \;x_{1eq}>\frac{2}{3}q_{z}$$

From (18), the Hessian is only positive if $\;x_{1eq}>\frac{2}{3}q_{z}$. Thus, the energy function has a local minimum at $\;x_{1eq}>\frac{2}{3}q_{z}$, which is the upper one-third region of the gap.

The stability analysis of the MEMS device can also be shown using linearization. The equilibria are determined by finding the solution so that the original state equations are zero, i.e., solving the following set of equations:

(19a) $${\dot{x}}_{1}=\frac{x_{2}}{m}=0$$

(19b) $${\dot{x}}_{2}=-k\left(x_{1}-q_{z}\right)-\frac{bx_{2}}{m}-\frac{x_{3}^{2}}{2A\epsilon }=0$$

(19c) $${\dot{x}}_{3}=\frac{-x_{1}x_{3}}{\epsilon RA}+\frac{u}{R}=0$$

Thus, we obtain:

(20a) $$x_{2}=0$$

(20b) $$x_{1}=q_{z}-\frac{x_{3}^{2}}{2Ak\epsilon }$$

(20c) $$x_{3}=\frac{u}{A\epsilon x_{1}\;}$$

Substituting (20c) into (20b) yields:

(21) $$x_{1}^{3}-q_{z}x_{1}^{2}+\frac{u^{2}}{c_{0}}=0;\;\;\;\;\;\;\;\;\;\;c_{0}=2k{\left(A\epsilon \right)}^{3}$$

There are three solutions for (21); one is negative for positive input voltages and can thus be ignored. The other two solutions are both positive. One of these equilibria is stable and the other is unstable. The stability of the system can also be analyzed by applying Lyapunov’s indirect method [32] to the system (4), which gives:

(22) $$A=\left[\begin{array}{ccc} 0 & \frac{1}{m} & 0\\ -k & -\frac{b}{m} & -\frac{x_{3}}{A\epsilon }\\ -\frac{x_{3}}{RA\epsilon } & 0 & -\frac{x_{1}}{RA\epsilon } \end{array}\right]$$

The eigenvalues of A are the solutions $\lambda_{i}$ of

(23) $$\left|\begin{array}{ccc} \lambda & -\frac{1}{m} & 0\\ k & \lambda +\frac{b}{m} & \frac{x_{3}}{A\epsilon }\\ \frac{x_{3}}{RA\epsilon } & 0 & \lambda +\frac{x_{1}}{RA\epsilon } \end{array}\right|=\lambda^{3}+\left(\frac{x_{1}}{RA\epsilon }+\frac{b}{m}\right)\lambda^{2}+\left(\frac{bx_{1}}{RA\epsilon m}+\frac{k}{m}\right)\lambda +\frac{kx_{1}}{RA\epsilon m}-\frac{x_{3}^{2}}{R{\left(A\epsilon \right)}^{2}m}=0$$

Substitute $-x_{3}^{2}=2kA\epsilon \left(x_{1}-q_{z}\right)$ from (20b) into (23) gives:

(24) $$\lambda^{3}+\left(\frac{x_{1}}{RA\epsilon }+\frac{b}{m}\right)\lambda^{2}+\left(\frac{bx_{1}}{RA\epsilon m}+\frac{k}{m}\right)\lambda +\frac{k}{RA\epsilon m}\left(3x_{1}-2q_{z}\right)=0$$

As all parameters, thus coefficients, are positive, the condition for negative eigenvalues is that $3x_{1}-2q_{z}>0,\;$ hence:

$$\;x_{1eq}>\frac{2}{3}q_{z}$$

i.e., the system is only stable within one third of its full gap and the region of stability is $x_{1}\;\in \left(\frac{2}{3}q_{z},q_{z}\right)$.

**Figure 2:**
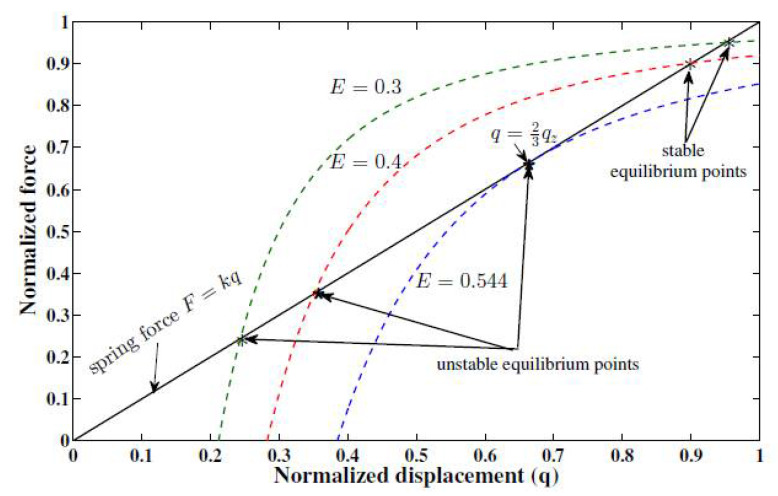
Pull-in displacement characteristic of the microactuator.

The explanation of the “pull-in” phenomenon is that the stability of the system at equilibrium is governed by two forces; the electrostatic force applied to the movable plate that pulls the plate down and the elastic stiffness (spring force) that pulls the spring, thus the top plate, up. Therefore, (14) is satisfied at the equilibrium. From (14) with the spring force constant k=1 and using (21), we can plot, as in Figure 2, the relationship between the spring force (straight line) and the electrostatic force (curved line), for various values of the input voltage *u*.

As shown, there are two intersections, which means two equilibria; one is stable, and one is unstable (saddle-node) [5]. When the upper plate’s deflection $q\;\left(x_{1}\right)$ is less than one-third of the zero voltage gap $q_{z}$, and with small perturbation of the gap, the actuator returns to its equilibrium point as the increase in the restoring force of the linear spring is greater than the increase in the electrostatic force. As $q\geq \frac{1}{3}q_{z}$, any perturbation will result in the attractive electrostatic force being the dominant force, hence causing the top plate to collapse to the fixed plate. For voltage values above the pull-in limit, there is no equilibrium point (the curves never intersect).

Due to the pull-in instability which is inherited behavior of voltage-controlled actuators, the equilibrium can be obtained once the desired stable position point ($x_{1d}=x_{1eq})\;$ is identified. For a given position $x_{1d}$ along with its corresponding given constant voltage ($V_{s}{}_{d}$), the charge is obtained as:

$$x_{3eq}=CV_{s}{}_{d}=\frac{A\epsilon }{x_{1eq}}V_{s}{}_{d}$$

In this paper, we are interested in the stabilization of the system over the entire gap position, i.e., the forced equilibrium

$$\left(x_{1eq},x_{2eq,}x_{3eq}\right)=\left(x_{1d},0,\frac{A\epsilon }{x_{1d}}V_{s}{}_{d}\right).\;$$

## 4. Design of the Controller and the Observer for the MEMS Actuator

### 4.1. The Port-Hamiltonian Model of the System

The port-Hamiltonian of dynamic systems can be defined as [19]:

$$\dot{x}=\left[J\left(x\right)-R\left(x\right)\right]\nabla H+g\left(x\right)u,$$

(25) $$y=g{\left(x\right)}^{T}\nabla H$$

where *x* $\in R^{n}$ is the states vector, *H* is the Hamiltonian function *H*:$\;R^{n}\to R$,  ∇H is the gradient vector of H, u is the control input of the system, J(X) is the interconnection matrix with $J\left(x\right)=-J{\left(x\right)}^{T}$, *R*(*x*) is the damping matrix with $R\left(x\right)=R{\left(x\right)}^{T}\geq 0$ and g(x) is the input matrix. The port-Hamiltonian model of the MEMS system (4) can be defined as [24]:

(26) $$\left[\begin{array}{c} {\dot{x}}_{1}\\ {\dot{x}}_{2}\\ {\dot{x}}_{3} \end{array}\right]=\left[\begin{array}{ccc} 0 & 1 & \;0\\ -1 & -b & \;0\\ 0 & 0 & -\frac{1}{R} \end{array}\right]\left[\begin{array}{c} \nabla_{x_{1}}H\\ \nabla_{x_{2}}H\\ \nabla_{x_{3}}H \end{array}\right]+\left[\begin{array}{c} 0\\ 0\\ \frac{1}{R} \end{array}\right]u$$

$$y=g{\left(x\right)}^{T}\nabla H.$$

Moreover, the Hamiltonian (energy function) of the system is defined as:

(27) $$H=\frac{1}{2}k{\left(x_{1}-q_{z}\right)}^{2}+\frac{x_{2}^{2}}{2m}+\frac{x_{1}x_{3}^{2}}{2A\epsilon }$$

### 4.2. The Controller Design Based on IDA-PBC Method

The main target of IDA-PBC is to find the control law u by matching the open-loop system (26) with the desired closed loop system which can be given by:

$$\dot{x}=\left[J_{d}\left(x\right)-R_{d}\left(x\right)\right]\nabla H_{d}$$

(28) $$y_{d}=g{\left(x\right)}^{T}\nabla H_{d}$$

The following can be designed to stabilize the system and to meet the required performance of the system: $J_{d}\left(x\right),R_{d}\left(x\right)\;$ and $H_{d}$ are the desired interconnection matrix, the desired damping matrix and the desired Hamiltonian, respectively. Here, we show the design of the controller based on [25], but we add coupling between the momentum state and the charge state via the interconnection matrix and state transformations as concluded in the following proposition:

**Proposition** **5.1.** *The electrostatic MEMS actuator model (26) can be controlled and stabilized at the equilibrium point (*$x_{1eq}$*, 0,*$\;x_{3eq}$*by the following dynamic control law u:*

(29) $$\begin{array}{c} u=\frac{R\Gamma }{x_{3}}\frac{-\left(\frac{\gamma x_{1}}{A\epsilon }+\gamma K_{1}\right)-\sqrt{{\left(\frac{\gamma x_{1}}{A\epsilon }+\gamma K_{1}\right)}^{2}+\frac{2}{A\epsilon }\left(z_{4}+\frac{x_{3}^{2}}{2A\epsilon }\right)}}{\frac{-1}{A\epsilon }}+\frac{R\gamma^{2}x_{1}x_{2}}{mx_{3}}+\frac{Rx_{2}\gamma^{2}K_{1}A\epsilon }{mx_{3}}\\ +\frac{RA\epsilon K_{i}x_{2}}{mx_{3}}+\frac{x_{3}x_{1}}{A\epsilon } \end{array}$$

$${\dot{z}}_{4}=-K_{i}\nabla_{z_{2}}H_{d},$$

with

$$\begin{array}{c} \Gamma =(-\gamma \frac{2x_{2}}{m}-K_{v}\frac{-\left(\frac{\gamma x_{1}}{A\epsilon }+\gamma K_{1}\right)-\sqrt{{\left(\frac{\gamma x_{1}}{A\epsilon }+\gamma K_{1}\right)}^{2}+\frac{2}{A\epsilon }\left(z_{4}+\frac{x_{3}^{2}}{2A\epsilon }\right)}}{\frac{-1}{A\epsilon }}\left(\frac{x_{1}}{A\epsilon }+K_{1}\right)\\ +\gamma x_{1}K_{v}\left(\frac{x_{1}}{A\epsilon }+K_{1}\right)+\gamma K_{1}A\epsilon K_{v}\left(\frac{x_{1}}{A\epsilon }+K_{1}\right), \end{array}$$

and the desired Hamiltonian of the system:

(30) $$H_{d}=\frac{1}{2}k{\left(z_{1}-q_{z}\right)}^{2}+\frac{z_{2}^{2}}{2m}+\frac{z_{1}z_{3}^{2}}{2A\epsilon }+\frac{1}{2}K_{1}z_{3}^{2}+\frac{1}{2}K_{i}{}^{-1}z_{4}^{2}$$

where $K_{i}>0$ is the integral term, and $K_{1}>0$ is a constant to stabilize the system. The port-Hamiltonian system of the closed loop system is given by:

(31) $$\left[\begin{array}{c} {\dot{z}}_{1}\\ {\dot{z}}_{2}\\ \begin{array}{c} {\dot{z}}_{3}\\ {\dot{z}}_{4} \end{array} \end{array}\right]=\left[\begin{array}{cc} \begin{array}{cc} \;0 & 1\\ -1 & -b \end{array} & \begin{array}{cc} \;0 & \;0\\ \;\gamma & \;K_{i} \end{array}\\ \begin{array}{cc} \;0\; & -\gamma \\ \;0 & -K_{i} \end{array} & \begin{array}{cc} -K_{v} & \;0\\ \;0 & \;0 \end{array} \end{array}\right]\left[\begin{array}{c} \nabla_{z_{1}}H_{d}\\ \nabla_{z_{2}}H_{d}\\ \begin{array}{c} \nabla_{z_{3}}H_{d}\\ \nabla_{z_{4}}H_{d} \end{array} \end{array}\right]\;$$

where γ is a constant coupling term between the momentum and the electric charge, being the essential difference between the interconnection matrix in this design and the interconnection matrix in [25]. The term $K_{v}>0$ is the damping term to asymptotically stabilize the system. The coordinate transformations are defined by:

$$z_{1}=x_{1};\;z_{2\;}=x_{2};$$

(32) $$z_{3}=\frac{-\left(\frac{\gamma z_{1}}{A\epsilon }+\gamma K_{1}\right)-\sqrt{{\left(\frac{\gamma z_{1}}{A\epsilon }+\gamma K_{1}\right)}^{2}+\frac{2}{A\epsilon }\left(z_{4}+\frac{x_{3}^{2}}{2A\epsilon }\right)}}{\frac{-1}{A\epsilon }}$$

**Proof of Proposition** **5.1.** The desired Hamiltonian function (30) can be chosen as a Lyapunov candidate. The time derivative of this function along the trajectories of (31) is given by:

(33) $$\begin{array}{c} {\dot{H}}_{d}=\nabla_{z_{1}}H_{d}{\dot{z}}_{1}+\nabla_{z_{2}}H_{d}{\dot{z}}_{2}+\nabla_{z_{3}}H_{d}{\dot{z}}_{3}+\nabla_{z_{4}}H_{d}{\dot{z}}_{4}\\ =\nabla_{z_{1}}H_{d}\nabla_{z_{2}}H_{d}+\nabla_{z_{2}}H_{d}\left(-\nabla_{z_{1}}H_{d}-b\nabla_{z_{2}}H_{d}+\gamma \nabla_{z_{3}}H_{d}+K_{i}\nabla_{z_{4}}H_{d}\right)\\ +\nabla_{z_{3}}H_{d}\left(-\gamma \nabla_{z_{2}}H_{d}-K_{v}\nabla_{z_{3}}H_{d}\right)+\nabla_{z_{4}}H_{d}\left(-K_{i}\nabla_{z_{2}}H_{d}\right)\\ =-b{\left|\nabla_{z_{2}}H_{d}\right|}^{2}-K_{v}{\left|\nabla_{z_{3}}H_{d}\right|}^{2}\\ =-b{\left|\frac{z_{2}}{m}\right|}^{2}-K_{v}{\left|\frac{z_{1}z_{3}}{A\epsilon }+K_{1}z_{3}\right|}^{2}\;\leq 0 \end{array}$$

which is negative semi-definite, i.e., the system is stable. Furthermore, asymptotic stability at the desired equilibrium can be verified by imposing the following detectability condition [33]:

***Condition 1:***

The output $y_{d}=\left[\begin{array}{c} \frac{z_{2}}{m}\\ \frac{z_{1}z_{3}}{A\epsilon }+K_{1}z_{3} \end{array}\right]$ of the closed-loop system (31) is detectable. □

Applying the detectability Condition 1 and given (33), asymptotic stability is guaranteed. The controller (29) is found using the transformation (32); that is, taking the derivate of the second transformation:

$${\dot{x}}_{2}={\dot{z}}_{2}$$

$$-\nabla_{x_{1}}H-b\nabla_{x_{2}}H=-\nabla_{z_{1}}H_{d}-b\nabla_{z_{2}}H_{d}+\gamma \nabla_{z_{3}}H_{d}+K_{i}\nabla_{z_{4}}H_{d}$$

$$-k\left(x_{1}-q_{z}\right)-\frac{x_{3}^{2}}{2A\epsilon }-\frac{bx_{2}}{m}=-k\left(z_{1}-q_{z}\right)-\frac{z_{3}^{2}}{2A\epsilon }-\frac{bz_{2}}{m}+\gamma \left(\frac{z_{1}z_{3}}{A\epsilon }+K_{1}z_{3}\right)+z_{4}$$

(34) $$z_{3}^{2}\left(\frac{-1}{2A\epsilon }\right)+z_{3}\left(\frac{z_{1}\gamma }{A\epsilon }+K_{1}\gamma \right)+\left(z_{4}+\frac{x_{3}^{2}}{2A\epsilon }\right)=0$$

This equation is quadratic in $z_{3}$ and the solution can be found by the general law of quadratic equations. By rearranging the third transformation and taking its derivate with respect to time we get:

$$z_{3}=\frac{-\left(\frac{\gamma z_{1}}{A\epsilon }+\gamma K_{1}\right)-\sqrt{{\left(\frac{\gamma z_{1}}{A\epsilon }+\gamma K_{1}\right)}^{2}+\frac{2}{A\epsilon }\left(z_{4}+\frac{x_{3}^{2}}{2A\epsilon }\right)}}{\frac{-1}{A\epsilon }}$$

$$\frac{z_{3}}{A\epsilon }=\frac{\gamma z_{1}}{A\epsilon }+\gamma K_{1}+\sqrt{{\left(\frac{\gamma z_{1}}{A\epsilon }+\gamma K_{1}\right)}^{2}+\frac{2}{A\epsilon }\left(z_{4}+\frac{x_{3}^{2}}{2A\epsilon }\right)}$$

$${\left(\frac{z_{3}}{A\epsilon }-\frac{\gamma z_{1}}{A\epsilon }-\gamma K_{1}\right)}^{2}={\left(\frac{\gamma z_{1}}{A\epsilon }+\gamma K_{1}\right)}^{2}+\frac{2}{A\epsilon }\left(z_{4}+\frac{x_{3}^{2}}{2A\epsilon }\right)$$

$$2\left(\frac{z_{3}}{A\epsilon }-\frac{\gamma z_{1}}{A\epsilon }-\gamma K_{1}\right)\left(\frac{{\dot{z}}_{3}}{A\epsilon }-\frac{\gamma {\dot{z}}_{1}}{A\epsilon }\right)=2\left(\frac{\gamma z_{1}}{A\epsilon }+\gamma K_{1}\right)\frac{\gamma {\dot{z}}_{1}}{A\epsilon }+\frac{2{\dot{z}}_{4}}{A\epsilon }+\frac{2x_{3}{\dot{x}}_{3}}{A^{2}\epsilon^{2}}$$

$$\frac{z_{3}{\dot{z}}_{3}}{A^{2}\epsilon^{2}}-\frac{\gamma {\dot{z}}_{1}z_{3}}{A^{2}\epsilon^{2}}-\frac{{\dot{z}}_{3}\gamma z_{1}}{A^{2}\epsilon^{2}}+\frac{\gamma^{2}z_{1}{\dot{z}}_{1}}{A^{2}\epsilon^{2}}-\frac{{\dot{z}}_{3}\gamma K_{1}}{A\epsilon }+\frac{{\dot{z}}_{1}\gamma^{2}K_{1}}{A\epsilon }=\frac{\gamma^{2}z_{1}{\dot{z}}_{1}}{A^{2}\epsilon^{2}}+\frac{{\dot{z}}_{1}\gamma^{2}K_{1}}{A\epsilon }+\frac{{\dot{z}}_{4}}{A\epsilon }+\frac{x_{3}{\dot{x}}_{3}}{A^{2}\epsilon^{2}}$$

(35) $$\gamma {\dot{z}}_{1}z_{3}+{\dot{z}}_{3}z_{3}-\gamma {\dot{z}}_{3}z_{1}-{\dot{z}}_{3}\gamma K_{1}A\epsilon ={\dot{z}}_{4}A\epsilon +x_{3}{\dot{x}}_{3}$$

$$-\gamma {\dot{z}}_{1}z_{3}+{\dot{z}}_{3}z_{3}-\gamma {\dot{z}}_{3}z_{1}-{\dot{z}}_{3}\gamma K_{1}A\epsilon ={\dot{z}}_{4}A\epsilon +x_{3}\frac{1}{R}\left(u-\frac{x_{3}x_{1}}{A\epsilon }\right)$$

$$\begin{array}{c} -\gamma \frac{z_{2}}{m}z_{3}-\frac{\gamma z_{2}z_{3}}{m}-z_{3}K_{v}\left(\frac{z_{1}z_{3}}{A\epsilon }+z_{3}K_{1}\right)+\frac{\gamma^{2}z_{1}z_{2}}{m}+\gamma z_{1}K_{v}\left(\frac{z_{1}z_{3}}{A\epsilon }+z_{3}K_{1}\right)+\frac{z_{2}\gamma^{2}K_{1}A\epsilon }{m}\\ +\gamma K_{1}A\epsilon K_{v}\left(\frac{z_{1}z_{3}}{A\epsilon }+z_{3}K_{1}\right)=-K_{i}\frac{z_{2}}{m}A\epsilon +x_{3}\frac{1}{R}\left(u-\frac{x_{3}x_{1}}{A\epsilon }\right) \end{array}$$

$$\begin{array}{c} z_{3}\left(-\gamma \frac{2x_{2}}{m}-K_{v}\left(\frac{x_{1}z_{3}}{A\epsilon }+z_{3}K_{1}\right)+\gamma x_{1}K_{v}\left(\frac{x_{1}}{A\epsilon }+K_{1}\right)+\gamma K_{1}A\epsilon K_{v}\left(\frac{x_{1}}{A\epsilon }+K_{1}\right)\right)+\frac{\gamma^{2}x_{1}x_{2}}{m}\\ +\frac{x_{2}\gamma^{2}K_{1}A\epsilon }{m}=-K_{i}\frac{x_{2}}{m}A\epsilon +x_{3}\frac{1}{R}\left(u-\frac{x_{3}x_{1}}{A\epsilon }\right) \end{array}$$

By rearranging and compensating the transformations, we obtain the control law (29).

**Remark** **5.1.** *By comparing the proposed method with that in [25], we notice that in this method we obtain a simpler control law and the transformation has been applied onto one coordinate (state): only on (x_3_). In [25], the transformation has been applied onto all states (x_1_, x_2_, x_3_)*.

**Remark** **5.2.** *By inspecting the controller (29), we notice the presence of the momentum (speed) state (due to a coupling which is another improvement) which is practically difficult to measure using special sensors [10]. In the following, we show the construction of the speed observer*.

### 4.3. The Design of the Speed Observer

Finally, the speed (v) in the MEMS actuator is difficult to measure, so a speed observer can be used to estimate the speed and then the momentum (the state in the port-controlled Hamiltonian formalism) is found using

$$p=x_{2}=mv.$$

The form of the speed observer (O) is taken from [10] which is:

(36) $$O=x_{2}-k_{2}x_{1},$$

where $k_{2}$ is a constant. We take the derivative of both sides of (36) with respect to time:

(37) $$\dot{O}={\dot{x}}_{2}-k_{2}{\dot{x}}_{1}$$

Substituting the terms from (4) into (37), yields:

(38) $$\dot{O}=-k\left(x_{1}-q_{z}\right)-\frac{b\left(O+k_{2}x_{1}\right)}{m}-\frac{x_{3}^{2}}{2A\epsilon }-k_{2}{\dot{x}}_{1}$$

The estimate of the O is formulated as

(39) $$\dot{\hat{O}}=-k\left(x_{1}-q_{z}\right)-\frac{b\left(\hat{O}+k_{2}x_{1}\right)}{m}-\frac{x_{3}^{2}}{2A\epsilon }-k_{2}{\dot{x}}_{1}$$

Finally, the estimate of the speed $x_{2}$ is given by:

(40) $${\hat{x}}_{2}=\hat{O}+k_{2}x_{1}$$

## 5. Numerical Simulations

The obtained theoretical results were validated through two numerical simulation environments: the MATLAB/Simulink environment was used to simulate the parallel plate MEMS actuator, and Multiphysics modelling software COMSOL (5.5) was used to simulate the radio frequency (RF) MEMS device.

### 5.1. Simulations Using MATLAB/Simulink environment

**Figure 3:**
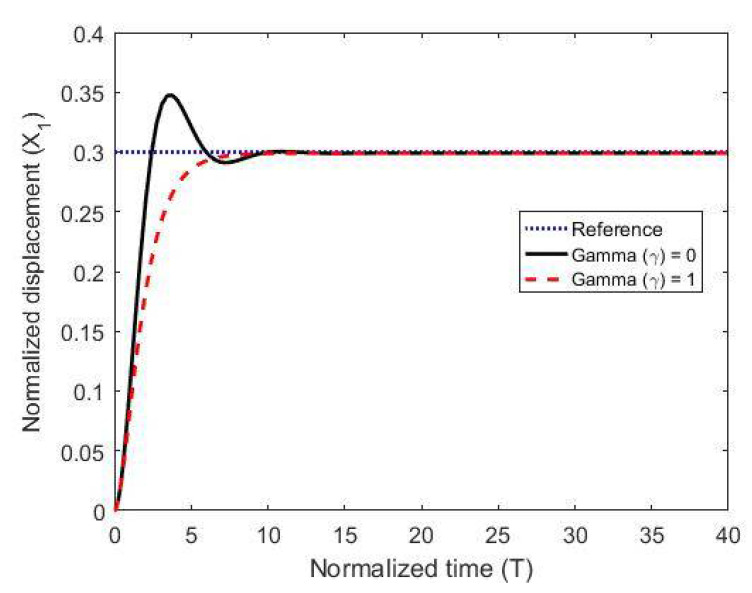
The normalized displacement with and without the coupling constant at a gap distance of 0.3.

**Figure 4:**
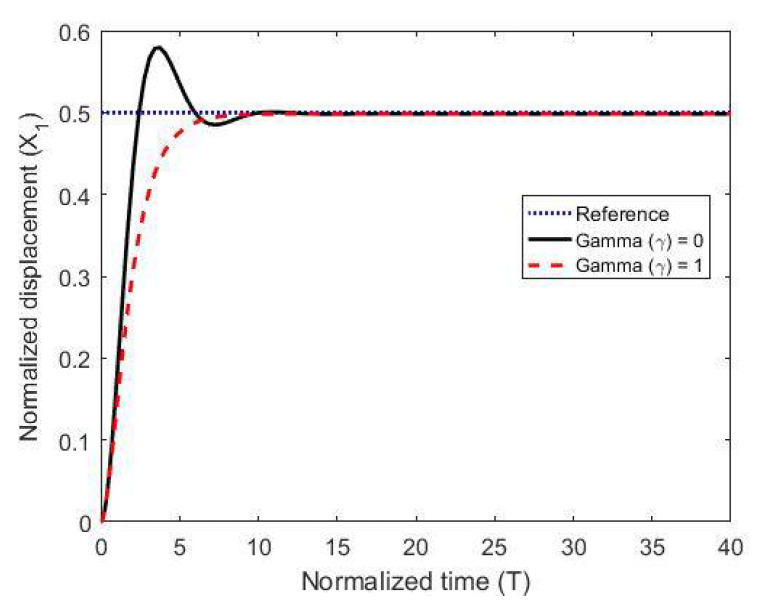
The normalized displacement with and without the coupling constant at a gap distance of 0.5.

**Figure 5:**
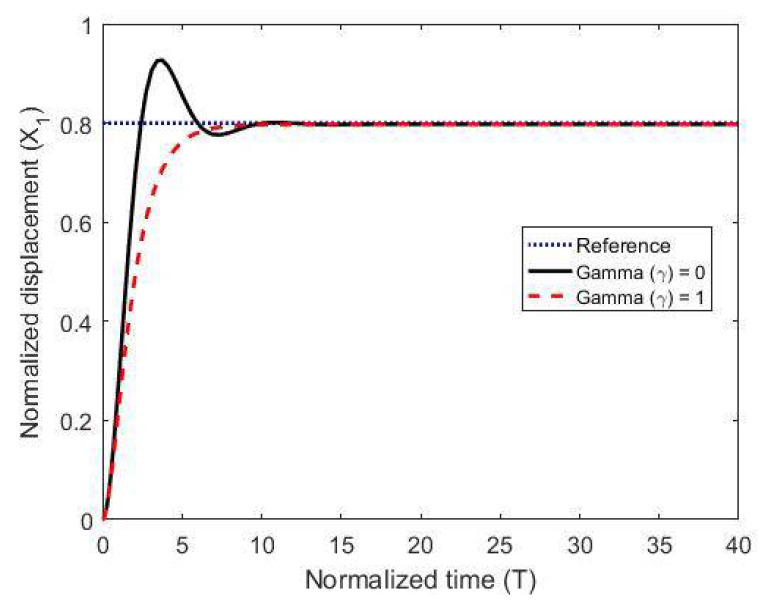
The normalized displacement with and without the coupling constant at a gap distance of 0.8.

To test the proposed controller, we built a numerical model using the MATLAB/Simulink environment and using normalized expressions. In these simulations, we chose the damping ratio ζ = 0.5, the natural frequency $\omega_{n}=1$, and the controller constants (gains) as $K_{1}=10,\;K_{v}=10,\;k_{2}=5$ and $K_{i}=0.003$. The results are shown in Figure 3, Figure 4 and Figure 5 for different reference signals and for a coupling term γ=1. To show the advantage of the proposed controller, the results are compared to the same model without the coupling constant, i.e., γ=0.

**Figure 6:**
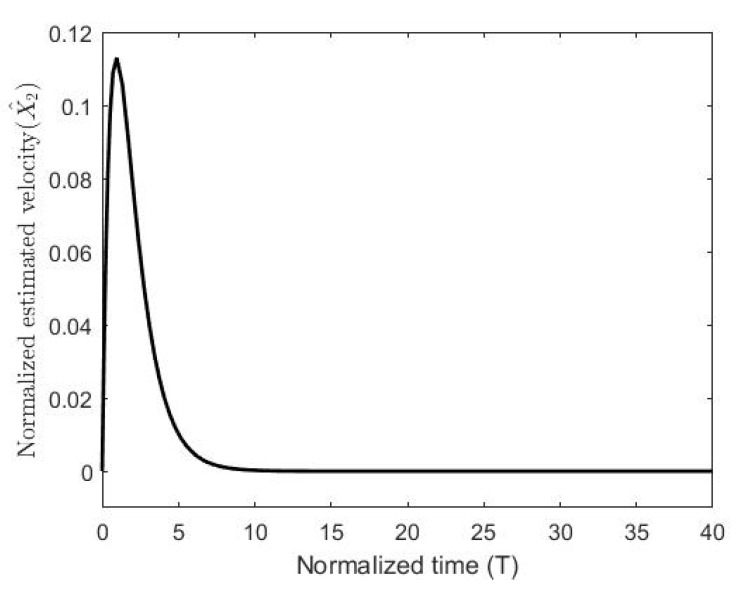
The normalized estimated velocity with the coupling constant at a gap distance of 0.3.

**Figure 7:**
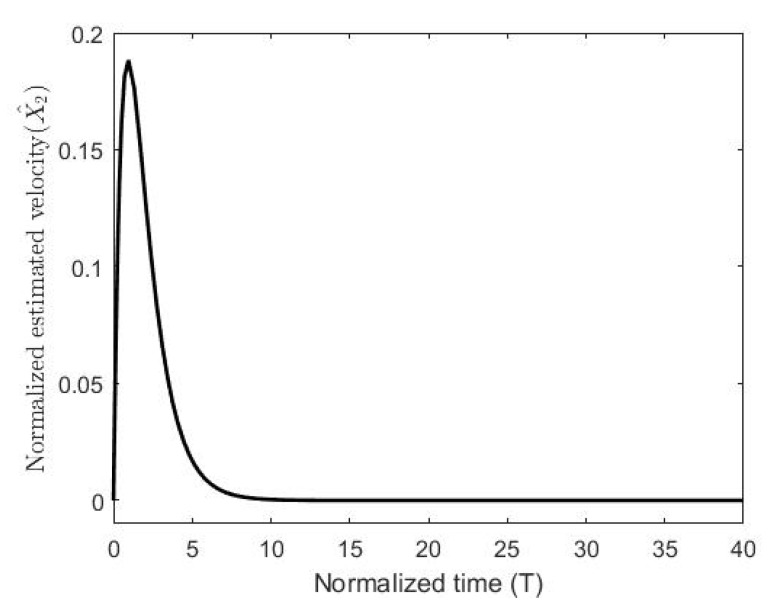
The normalized estimated velocity with the coupling constant at a gap distance of 0.5.

**Figure 8:**
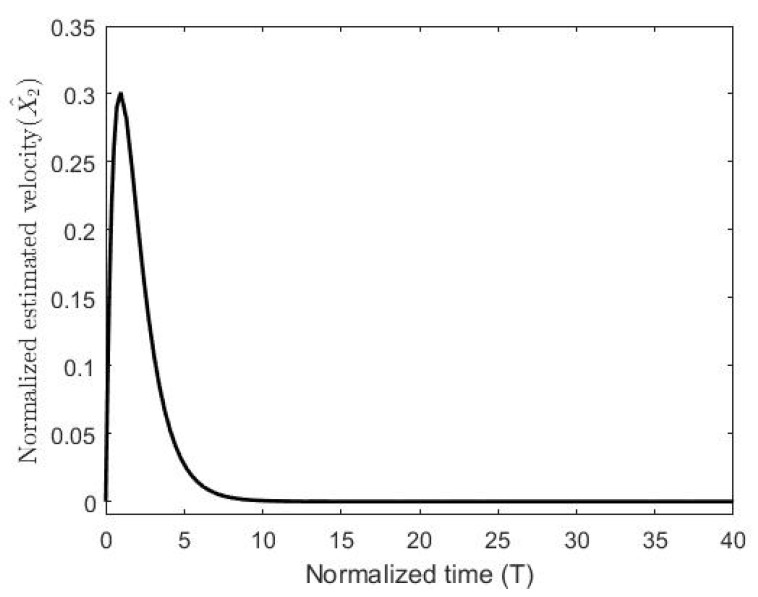
The normalized estimated velocity with the coupling constant at a gap distance of 0.8.

The figures demonstrate that both controllers stabilize the system throughout the full gap distance, i.e., beyond the pull-in range. Also, the coupling improves the response by reducing the overshoot for a step response. The estimated speed is shown in Figure 6, Figure 7 and Figure 8 for different reference signals and for γ=1.

**Figure 9:**
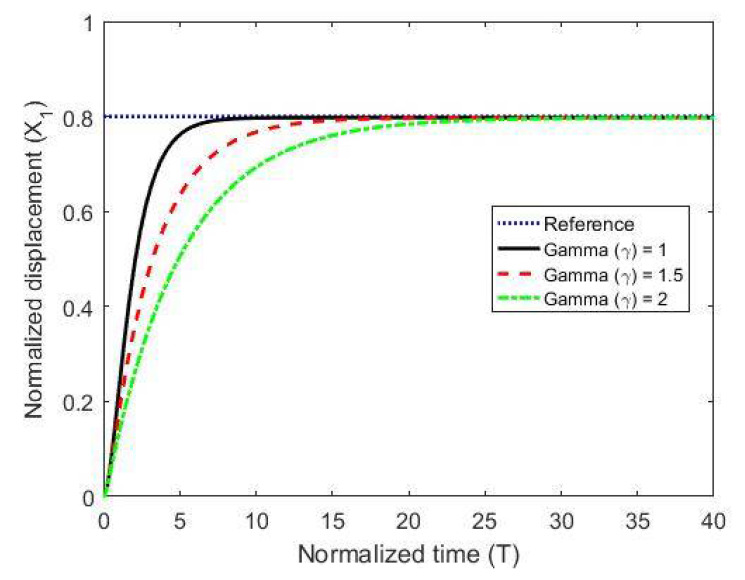
The normalized displacement with a different coupling term at a gap reference of 0.8.

The results for stabilization of the plate by varying the coupling term (γ) are shown in Figure 9.

**Figure 10:**
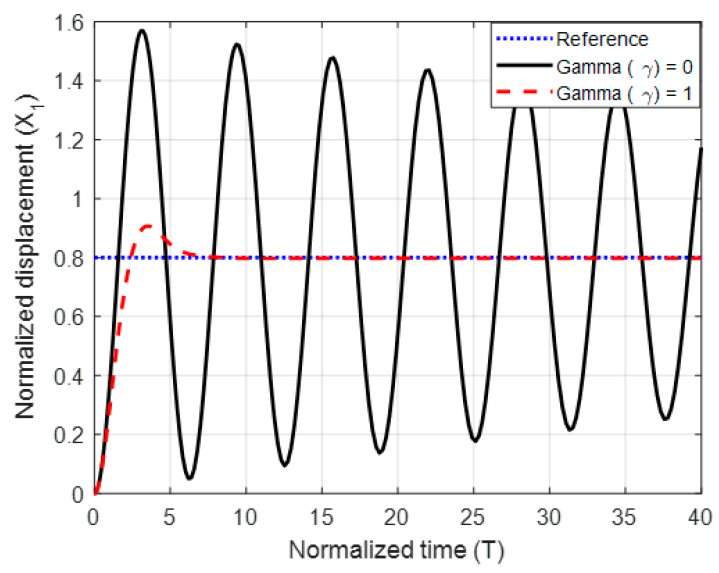
The normalized displacement with and without the coupling constant at a gap distance of 0.8 and damping ratio ζ = 0.01.

**Figure 11:**
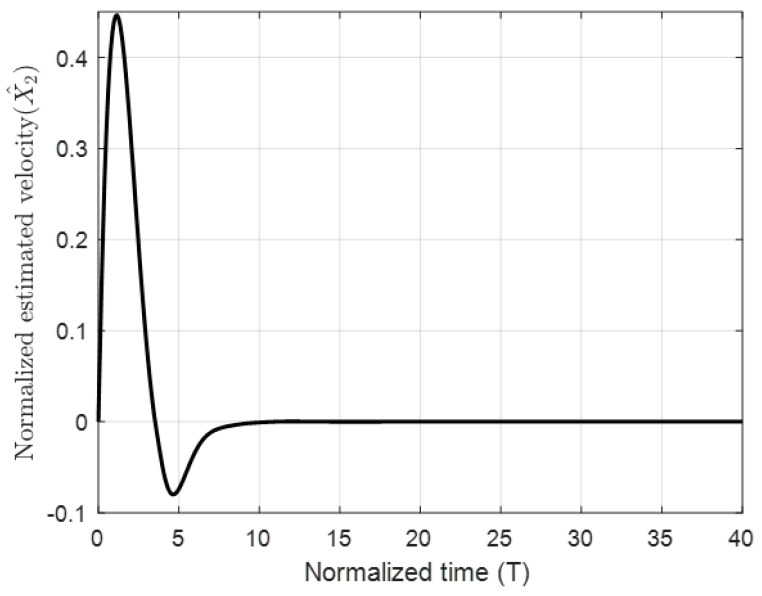
The normalized estimated velocity with the coupling constant at a gap distance of 0.8 and damping ratio ζ = 0.01.

**Figure 12:**
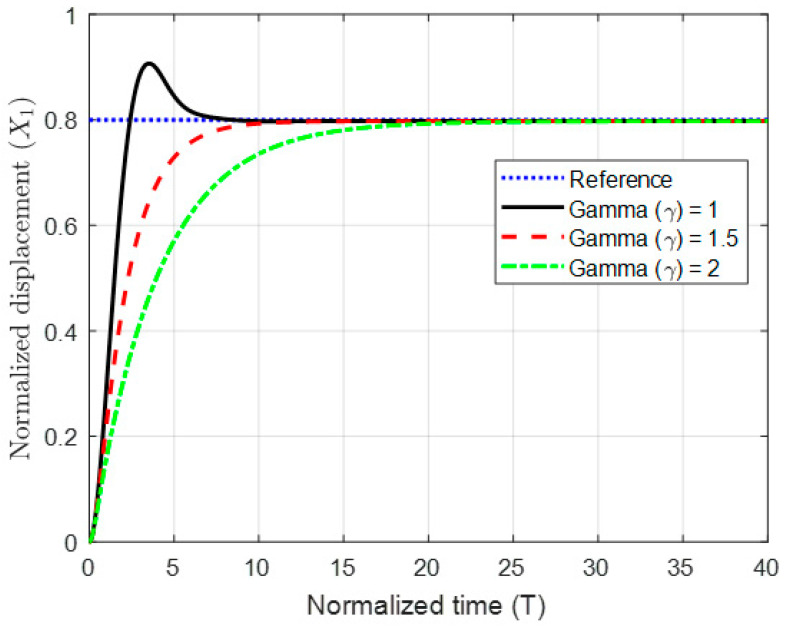
The normalized displacement with different coupling terms at a gap reference of 0.8 and damping ratio ζ = 0.01.

Finally, to show the advantage and robustness of this method even if the system has a lighter damping factor, the closed-loop system has been simulated with a damping factor of ζ = 0.01 as shown in Figure 10, Figure 11 and Figure 12.

The figures show that without coupling the system suffers from oscillation and overshoot above its normalized limit ($x_{1}=1$). The transient behavior of the system has been significantly improved by introducing the coupling, which demonstrates the effectiveness of the proposed controller.

### 5.2. Simulations Using Multiphysics Modelling Software COMSOL

**Figure 13:**
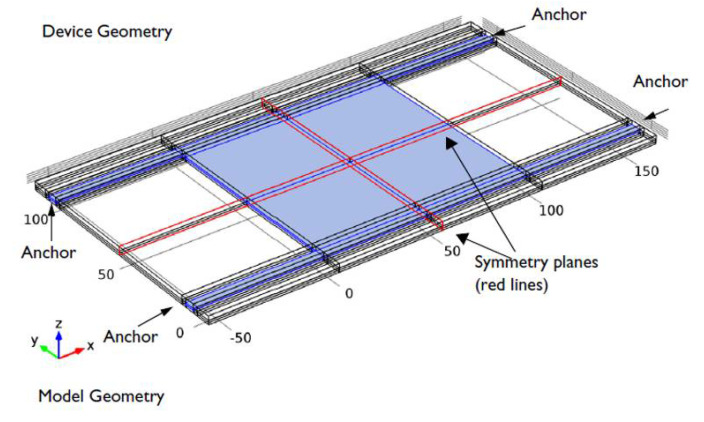
Schematic view of the RF MEMS device.

**Figure 14:**
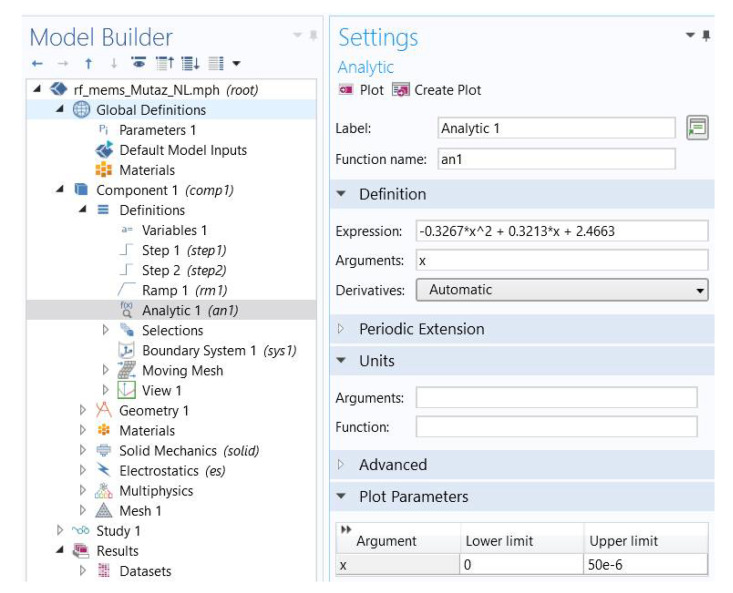
Model configuration and setting on COMSOL.

The results have also been demonstrated with numerical simulations on the RF MEMS device using COMSOL software. We use the model example of [34] to illustrate our simulation results. The schematic view of the RF MEMS device is shown in Figure 13. The device consists of square parallel plates (electrodes), one grounded (fixed) and one movable plate formed from polysilicon and suspended 0.9 μm above a 0.1 μm thick dielectric layer of silicon nitride. The device is actuated electrostatically by applying a voltage signal. The parameters of the model are the same as in [34], and are repeated here in Table 1 for ease of reference:

For the sake of controller design aiming at producing the voltage input, the RF model has been first simulated in the MATLAB/Simulink environment. We used the “simout” function, which allows the definition of a voltage signal as an expression from a set of points either with linear interpolation (best fit line) or as a polynomial formula. This formula is introduced to the COMSOL model as depicted in Figure 14.

**Figure 15:**
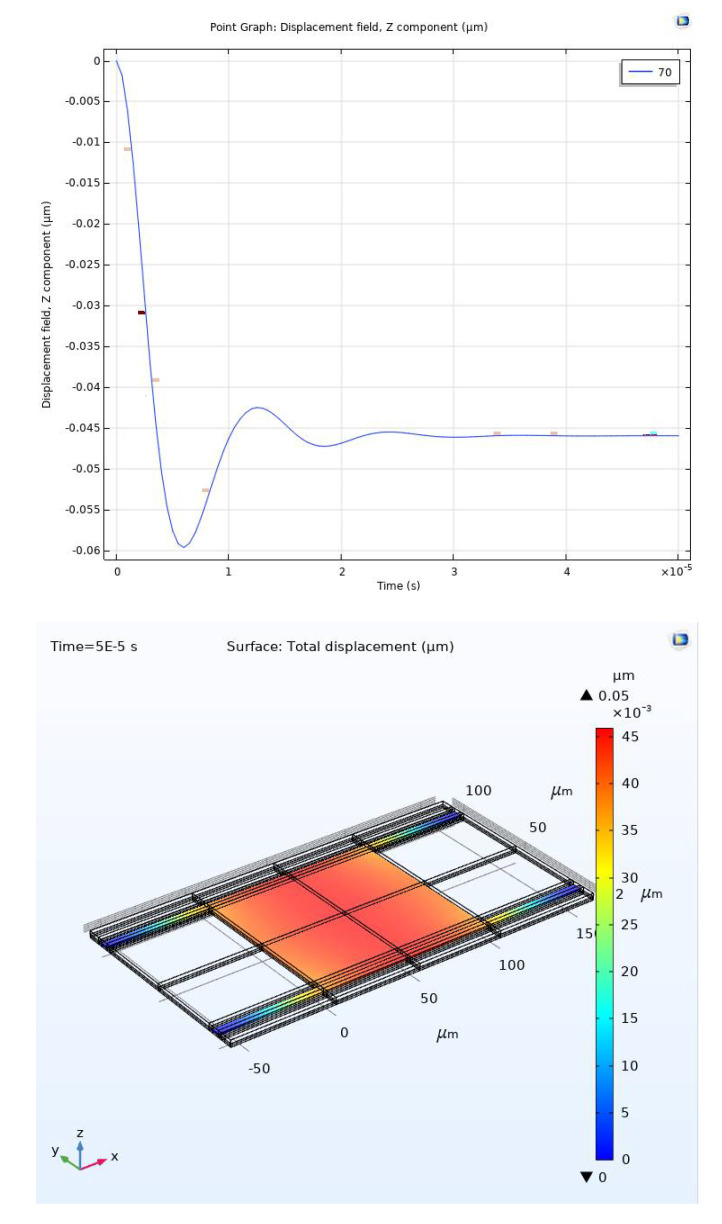
Stabilization at the first third of the full gap: top graph (1-D), bottom graph (3-D).

**Figure 16:**
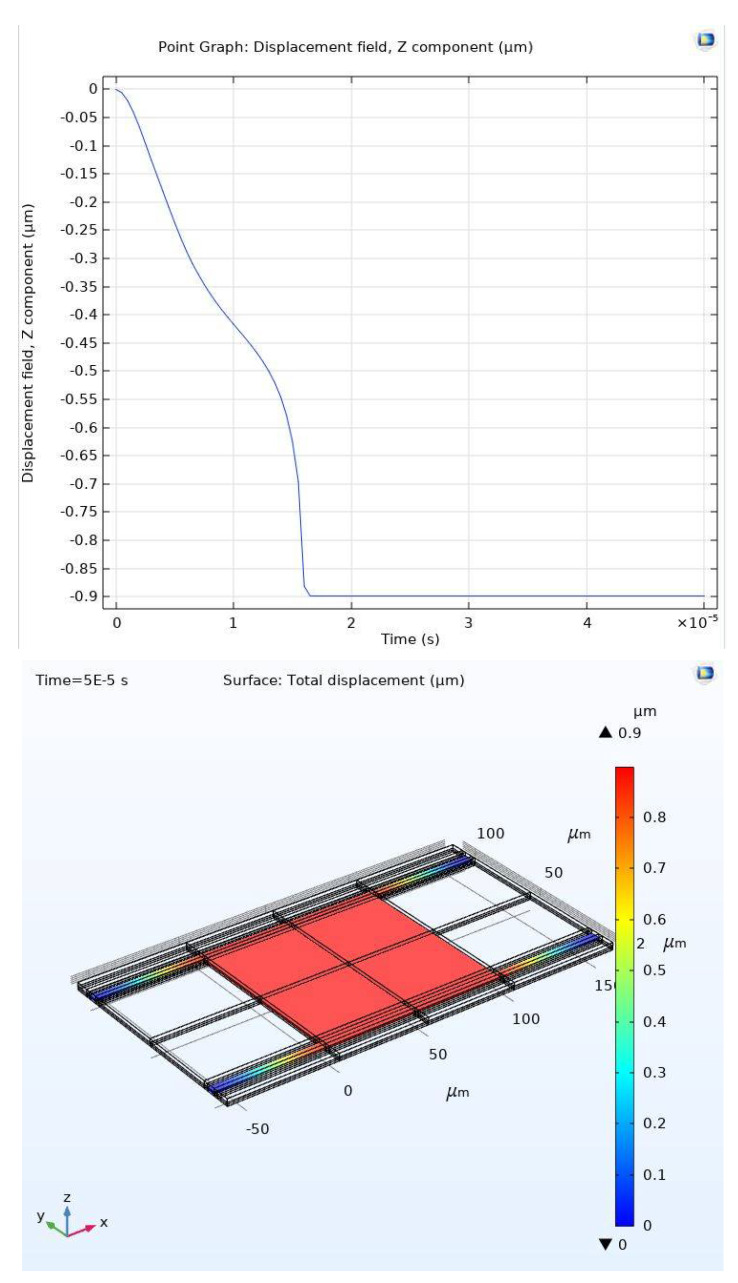
Stabilization at the last third of the full gap: top graph (1-D), bottom graph (3-D).

Figure 15 and Figure 16 show the simulation results for the RF MEMS device using COMSOL software. Simulation results are obtained for displacements through the gap (0–1.01 μm). Figure 15 shows the stabilization of the upper (movable) plate at the first one-third of the gap at 0.046 μm.

Figure 16 shows the stabilization of the movable plate at 0.9 μm which is in the last third of the full gap, i.e., beyond the pull-in instability range. Thus, the effectiveness of the proposed controller is demonstrated by two sets of simulations, which both showed successful stabilizing of the electrostatically MEMS device through the full range with improved performance.

**Figure 17:**
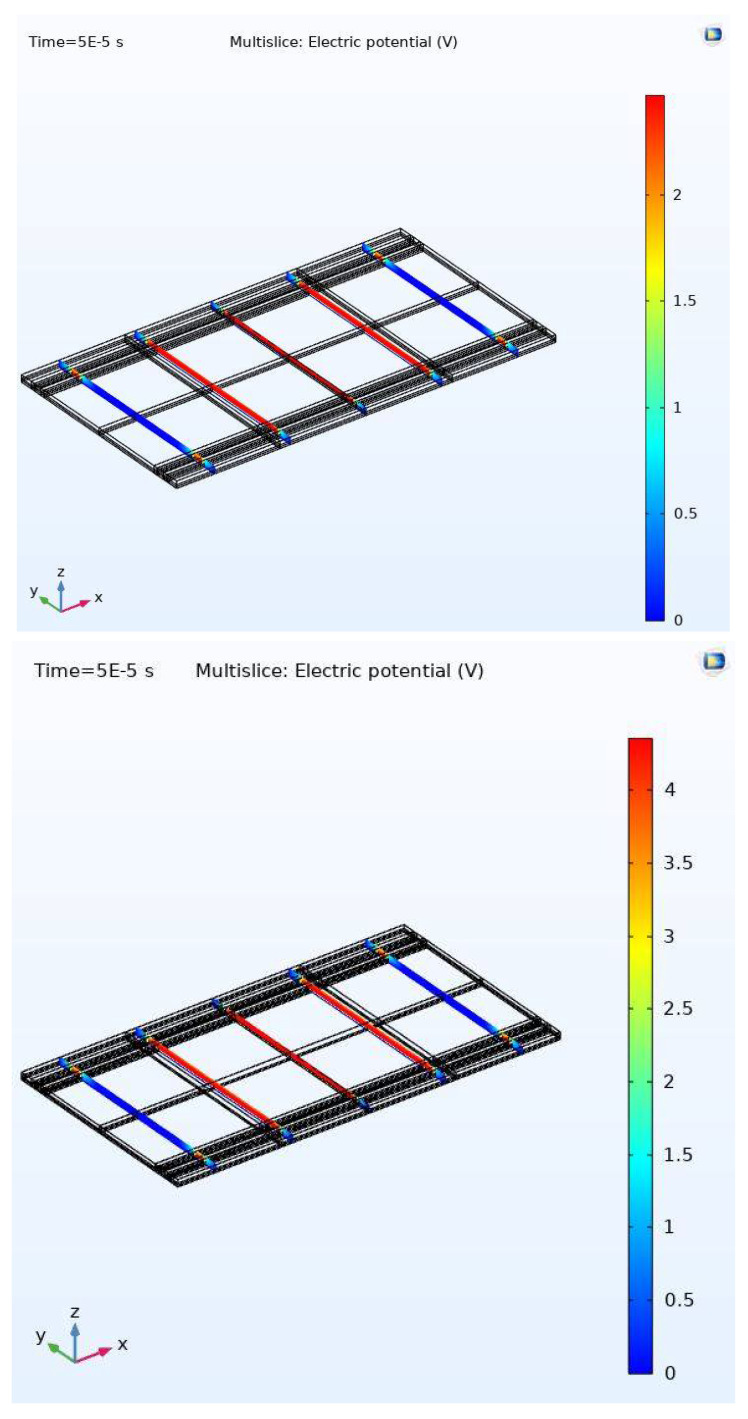
Control input (voltage) at two positions: top graph (0.046 μm), bottom graph (0.9 μm ).

Finally, Figure 17 shows the voltage (electric potential (V)) as the control input that actuates the RF MEMS device and moves the upper plate in the z coordinate towards the fixed plate (for the 2 positions above). As expected from the theory, a higher voltage signal (around 4.5 V) was needed to move the plate to the 0.9 μm position (near the fixed plate) compared to around 2.5 V that was needed to stabilize the movable plate at 0.046 μm (i.e., in the first-third of gap).

## 6. Conclusions

In this paper, the IDA-PBC method was used to design a dynamic controller for stabilization and improving the performance of an electrostatic MEMS actuator across the full gap distance. An improved response was obtained by utilizing coordinate transformations and forcing a coupling between the electric charge and the momentum in the interconnection matrix. A speed observer was also employed to estimate the speed (momentum), which is hard to measure in real applications. Simulation results of the electrostatic MEMS actuator suitably verified the proposed dynamic IDA-PBC controller and the speed observer. The validation of the proposed control design method was obtained using two simulation environments, namely: MATLAB/Simulink and COMSOL Multiphysics.

## Figures and Tables

**Table 1:** Parameters of the RF MEMS device.

Parameter	Value
Plate length	*L* = 50 μm
Plate thickness	t = 2 μm
Gap between the plates	$q_{z}=1.1\;\mu m=1.1\;\mu m$
Permittivity	$\epsilon =4.5=4.5^{{}^{\circ}}$
Density	$\rho =2320\;\mathrm{kg}/m^{3}/m^{3}$
Damping ratio	*ζ* = 1 × $10^{-6}$
Spring stiffness	*k* = 128 N/m
